# The Effect of Assisted Reproductive Technologies on Human Sex Ratios

**DOI:** 10.1111/ajo.13940

**Published:** 2025-01-26

**Authors:** Ektoras X. Georgiou, Yossi Mizrachi, Rebecca Kelley, Sharyn Stock‐Myer, John Stevens, David K. Gardner, Alex Polyakov

**Affiliations:** ^1^ Reproductive Services Unit The Royal Women's Hospital Parkville Australia; ^2^ Melbourne IVF East Melbourne Victoria Australia; ^3^ Department of Obstetrics, Gynaecology, and Newborn Health University of Melbourne Parkville Australia; ^4^ Virtus Diagnostics East Melbourne Victoria Australia

**Keywords:** ART, blastocyst, IVF, PGT‐A, sex ratio

## Abstract

**Background:**

Modern assisted reproductive technology (ART), including pre‐implantation genetic testing for aneuploidy (PGT‐A), has opened new avenues in understanding early embryonic events and has simultaneously raised questions about the impact of ART itself on sex ratios.

**Aims:**

The primary aim was to investigate whether patient demographic characteristics, ovarian stimulation protocols or laboratory characteristics in ART influence sex ratios. The secondary aim was to relate the blastocyst sex ratio (BSR) to the corresponding secondary sex ratio (SSR) in our patient cohort.

**Materials and Methods:**

We conducted a retrospective single‐centre cohort study on consecutive PGT‐A cycles from January 2019 to February 2022. We analysed demographic data, stimulation protocols, laboratory characteristics and pregnancy outcomes.

**Results:**

The euploidy rate was 45.1% (2608/5777), BSR 1.07 and euploid BSR 1.03. There was no statistical difference in the ploidy status of XX and XY blastocysts and on multivariate logistic regression analysis, there was no association between euploidy and BSR or any other variables examined, including female age and stimulation protocol. The SSR was 1.02 (1294 embryo transfers) with no statistical difference in biochemical pregnancy (*p* = 0.41), clinical pregnancy (*p* = 0.56), miscarriage (*p* = 0.65) or live birth rates (*p* = 0.40) based on embryo sex.

**Conclusions:**

Our study indicates that the euploid BSR is not skewed by sex, and there is no relationship between BSR and euploidy, patient characteristics or stimulation protocols. Pregnancy outcomes did not vary by sex, and the SSR was consistent with the SSR of the general Australian population at birth.

## 1 Introduction

The term sex ratio refers to the ratio of males to females in a population, and more specifically, primary sex ratio (PSR) refers to the ratio at fertilisation and secondary sex ratio (SSR) refers to the ratio at birth. The World Health Organization estimated the SSR to be 1.011 in 2021, indicating that globally, more males are born than females [1]. Apart from its biological significance in terms of long‐term survival of the human race, the SSR is an important demographic and social indicator. Potential deviations to the SSR, as occurred with sex‐selective abortion practices, could have significant implications on societal function [2, 3].

Historically, it has been easier to study the SSR; however, assisted reproductive technology (ART) coupled with preimplantation genetic testing for aneuploidy (PGT‐A) offers the scientific community novel opportunities to understand early embryological events with respect to sex ratios and their relationship, if any, with aneuploidy. In spontaneous conception, any discrepancy between the PSR and SSR is accounted for by pregnancy loss, which may occur naturally (e.g., miscarriage) or by iatrogenic means (e.g., termination of pregnancy).

Despite the fact that X and Y spermatozoa are essentially produced in a 1:1 ratio [4], the PSR is thought to be > 1, although the reasons behind this remain elusive [5]. Further, historically, it is accepted that the PSR is greater than the SSR [5] and several endogenous and exogenous factors are thought to underlie this finding [6], including behavioural patterns and environmental toxins [7].

Several studies have reported that the SSR arising from ART does not differ significantly from the overall population SSR [8, 9]. This observation is supported by a recent retrospective study using the UK Human Fertilisation and Embryology Authority (HFEA) database and incorporating over 1 million cycles, which reported the SSR arising from ART cycles (1.04) to be comparable to the overall national SSR rate (1.053) [10]. However, the picture changes somewhat when specific ART procedures are examined more closely, for example, intracytoplasmic sperm injection (ICSI) versus conventional IVF [11, 12, 13, 14]. An impact on sex ratios has also been reported with blastocyst transfer as compared to cleavage‐stage transfer [9, 13, 14, 15], although not consistently so [16]. Laboratory variables, in particular atmospheric O_2_, have also been shown to affect sex ratios, something which is not observed when the culture of embryos occurs at 5% O_2_ [17]. In addition, patient variables, such as smoking [18] and diet [19], could potentially influence sex ratios.

More recently, the concept of a higher PSR as compared to the SSR has been challenged by Orzack et al. [20] who examined 139 704 embryos by fluorescence in situ hybridization or array comparative genomic hybridization between Days 3 and 6 and found very weak, if any, evidence of sex preponderance (sex ratio 1.01). It should be noted that the PSR is difficult to determine definitively, with the closest current option being cleavage or, in modern practice, blastocyst stage biopsy (blastocyst sex ratio, BSR).

Given these conflicting reports in the literature and the ever‐increasing use of ART globally [21], the primary aim of our study was to investigate whether patient demographic characteristics, ovarian stimulation protocols or laboratory characteristics in ART influence the overall and euploid BSRs. The secondary aim of the study was to relate the BSR to the corresponding SSR in our patient cohort.

## 2 Materials and Methods

### 2.1 Study Design

This was a retrospective single‐centre cohort study incorporating all consecutive PGT‐A cycles that were carried out between January 2019 and February 2022. The study protocol was prospectively approved by the Melbourne IVF Human Research Ethics Committee (HREC Ref: 92/22). The outcomes examined as part of our primary outcome were the overall and euploid BSRs and their relationship to:

– Demographic characteristics: female age, male age, female BMI, AMH, female smoking status

– Ovarian stimulation protocols: stimulation protocol (antagonist, long agonist, flare), type of gonadotrophin (FSH, FSH + LH), total gonadotrophin dose, trigger (hCG, GnRH agonist), days of stimulation

– Laboratory characteristics: fertilisation method (IVF, ICSI)

For the secondary aim, we included patients delivering up to and including 31 December 2021. The secondary outcomes assessed were biochemical pregnancy rate, clinical pregnancy rate, miscarriage rate and live birth rate, as defined by Zegers‐Hochschild et al. [22]. Relevant IVF variables (endometrial thickness, embryo developmental stage and quality) and obstetric outcomes (gestational age, mode of birth and birth weight) were also examined.

The inclusion criteria were (1) blastocyst biopsy for PGT‐A from embryos created through either IVF or ICSI and (2) single embryo transfer. The exclusion criteria were (1) PGT‐M or PGT‐SR cycles, (2) gamete donation cycles, (3) double embryo transfer and (4) fresh embryo transfer without PGT‐A.

The indications for PGT‐A included advanced maternal age, previous poor IVF outcomes such as implantation failure, recurrent pregnancy loss and patient request.

### 2.2 Ovarian Stimulation, Culture and Fertilisation Protocols

Patients underwent ovarian stimulation using standard protocols (antagonist, long agonist or flare) according to treating clinician preference with recombinant follicle‐stimulating hormone (FSH) or gonadotrophins with dual FSH and luteinising hormone (LH) activity (recombinant or human menopausal gonadotrophins). Patients received either human chorionic gonadotrophin (hCG) (Ovidrel, Merck Serono, Australia) or gonadotrophin‐releasing hormone (GnRH) agonist (Decapeptyl, Ferring Pharmaceuticals, Australia) trigger and transvaginal ultrasound‐guided oocyte retrieval took place under sedation approximately 36 h thereafter.

Ejaculated semen samples were held at room temperature for up to 1 h to liquefy before processing. Frozen sperm samples were thawed for 10 min in a water bath at room temperature. Sperm was prepared using a density gradient (40% and 80%, PureSperm, NidaCon, Sweden) and centrifugation at 300 *g* for 10 min. Samples were then washed in G‐IVF PLUS (Vitrolife, Sweden), centrifuged and resuspended in G‐IVF PLUS and incubated at 37°C, 6% CO_2_ until insemination.

Cumulus–oocyte complexes were transferred into G‐IVF PLUS and cultured in a benchtop incubator (MINC, Cook Medical, Australia or BT‐37, CooperSurgical, Denmark) at 37°C, 6% CO_2_ and 5% O_2_.

Oocytes destined for conventional IVF were placed in groups of ≤ 4 in 0.5 mL G‐IVF PLUS and approximately 10^5^ motile sperm were added per well.

Oocytes were incubated overnight in benchtop incubators at 37°C, under 5% O_2_ and 6% CO_2_. The next day, the cumulus from inseminated oocytes was removed by pipetting 16–18 h post insemination.

Oocytes destined for ICSI were denuded > 1 h post oocyte retrieval by < 30 s exposure to 40 IU hyadase (CooperSurgical) followed by pipetting and washing in G‐IVF PLUS. MII oocytes were injected with sperm in GIVF, and inseminated oocytes were cultured in an Embryoscope or Embryoscope+ incubator (Vitrolife) under 5% O_2_ and 6% CO_2_ in G‐TL (Vitrolife).

Oocytes were checked for normal fertilisation at 16–18 h post insemination using EmbryoViewer software. On Day 5 of culture, the medium was refreshed with pre‐equilibrated G‐TL.

### 2.3 Blastocyst Biopsy and PGT‐A

Trophectoderm biopsy was performed on Days 5–7 of embryo development followed by PGT‐A using next generation sequencing using the MiSeq platform (Veriseq PGS, Vitrolife). Biopsied embryos were vitrified using the RapidVit Blast and Rapid‐I device (Vitrolife) according to the manufacturer's protocol. Embryos were labelled as euploid (no chromosome abnormalities detected), aneuploid (whole or segmental aneuploidy detected) or mosaic (intermediate copy number in clean profiles: mosaicism called in the range 30%–70% of chromosome level).

### 2.4 Embryo Transfer

The endometrium was prepared for transfer per clinician preference and patient characteristics (natural cycle, artificial cycle or FSH thaw). Blastocysts were warmed using RapidWarm Blast (Vitrolife). Single embryo transfer of a euploid thawed embryo in EmbryoGlue (Vitrolife) was performed under transabdominal ultrasound guidance using a Guardia AccessET catheter (Cook Medical) or Wallace Sure Pro Ultra catheter (CooperSurgical).

### 2.5 Statistical Analysis

Statistical analysis was performed with SPSS (v28) (IBM, USA). Sex ratios are expressed as the ratio of males to females. Continuous variables are expressed as mean ± standard deviation and were compared using Student's *t*‐test. Categorical variables are presented as percentages with 95% confidence intervals and were compared using the chi‐squared test. A multivariate logistic regression analysis was performed to identify any independent parameters associated with an XY karyotype; variables were chosen on the basis of their potential impact on sex ratios. *p* < 0.05 was considered statistically significant.

## 3 Results

We included 5811 embryos arising from 1981 PGT‐A cycles from 1352 patients. Thirty‐four embryos were excluded from further analysis because of an indeterminate sex chromosome analysis. The BSR was skewed in favour of male sex at 1.07, with 51.7% of embryos (95% CI: 50.4%–53.0%) diagnosed as male and 48.3% (95% CI: 47.1%–49.6%) as female (Table 1). The BSR for embryos formed by IVF followed a similar pattern, with a sex ratio of 1.12 (male 52.9% [95% CI: 50.9%–55.0%], female 47.1% [95% CI: 45.1%–49.1%]) (Table 1). The BSR for embryos formed by ICSI was 1.03 and was balanced between the sexes (male 50.8% [95% CI: 49.1%–52.5%], female 49.2% [95% CI: 47.5%–50.9%]) (Table 1). PGT‐A testing demonstrated a total euploidy rate of 45.1% (2608/5777), aneuploidy rate of 49.6% (2849/5777) and mosaicism of 5.5% (320/5777).

**TABLE 1:** Sex ratios at different stages of embryo development.

	Female proportion (*n*/total)	Female % (95% CI)	Male proportion (*n*/total)	Male % (95% CI)	Sex ratio
Blastocysts formed by ICSI	1692/3439	49.2% (47.5%–50.9%)	1747/3439	50.8% (49.1%–52.5%)	1.03
Blastocysts formed by IVF	1101/2338	47.1% (45.1%–49.1%)	1237/2338	52.9% (50.9%–55.0%)	1.12
Blastocysts	2793/5777	48.3% (47.1%–49.6%)	2984/5777	51.7% (50.4%–53.0%)	1.07
Euploid blastocysts	1285/2608	49.3% (47.3%–51.2%)	1323/2608	50.7% (47.8%–52.7%)	1.03
Thawed	630/1319	47.8% (45.0%–50.5%)	689/1319	52.2% (49.5%–54.9%)	1.09
Survived	617/1294	47.7% (44.9%–50.4%)	677/1294	52.3% (49.65%–55.1%)	1.10
Transferred	590/1232	47.9% (45.1%–50.7%)	642/1232	52.1% (49.3%–54.9%)	1.09
Implantation	349/744	46.9% (43.3%–50.6%)	395/744	53.1% (49.4%–56.7%)	1.13
Miscarriage	47/86	54.7% (43.5%–65.4%)	39/86	45.3% (34.6%–56.5%)	0.83
Live birth	223/451	49.4% (44.7%–54.2%)	228/451	50.6% (45.8%–55.3%)	1.02

### 3.1 Baseline, Ovarian Stimulation and Laboratory Characteristics

There was no statistical difference in the ploidy status of XX and XY blastocysts (*p* = 0.19) (Table 2), with a euploid BSR of 1.03. Baseline characteristics, in terms of female age, male age, female BMI, AMH and female smoking status, did not differ significantly according to blastocyst sex (Table 2). The choice of stimulation protocol, gonadotrophin choice, daily dose, total dose and trigger did not differ according to blastocyst sex (Table 2). Overall, the ICSI rate was 59.5% (3439/5777) and there was no difference noted based on fertilisation method (Table 2). On multivariate logistic regression analysis, there was no association between euploidy and BSR or any potential confounders (Table 3).

**TABLE 2:** The relationship between XX and XY embryos to ploidy, baseline characteristics, ovarian stimulation and fertilisation method. Values are expressed as mean ± standard deviation.

	XX (*n* = 2793)	XY (*n* = 2984)	*p*
PGT‐A results			
Euploid	1285 (46.0%)	1323 (44.3%)	0.19
Aneuploid	1345 (48.2%)	1504 (50.4%)	
Mosaic	163 (5.8%)	157 (5.3%)	
Female age	38.1 ± 3.4	38.1 ± 3.4	0.69
Male age	39.2 ± 5.2	39.1 ± 5.2	0.82
Female BMI (kg/m^2^)	25.0 ± 5.4	24.9 ± 5.3	0.71
AMH (pmol/L)	19.5 ± 11.3	20.8 ± 9.9	0.58
Smoker	99 (3.5%)	101 (3.4%)	0.77
Stim protocol			
Antagonist	2602 (93.2%)	2770 (92.8%)	0.74
Long agonist	118 (4.2%)	126 (4.2%)	
Flare	73 (2.6%)	88 (2.9%)	
Gonadotropins			
FSH only	2383 (85.3%)	2589 (86.8%)	0.11
FSH + LH	410 (14.7%)	395 (13.2%)	
Total gonadotropin dose (IU/mL)	2252 ± 1153	2203 ± 1069	0.09
Trigger type			
HCG	2006 (71.8%)	2156 (72.3%)	0.75
GnRH agonist	787 (28.2%)	828 (27.7%)	
Length of stim (days)	8.7 ± 3.2	8.6 ± 3.0	0.07
ICSI	1692 (60.6%)	1747 (58.5%)	0.11

Abbreviations: AMH, anti‐Mullerian hormone; FSH, follicle stimulating hormone; GnRH, gonadotrophin‐releasing hormone; HCG, human chorionic gonadotrophin; ICSI, intracytoplasmic sperm injection; LH, Luteinising hormone; PGT‐A, pre‐implantation genetic testing for aneuploidy.

**TABLE 3:** Multivariate logistic regression analysis for euploid embryos and potential confounders.

	AOR (95% CI)	*p*
XX karyotype (XY set as reference)	1.84 (0.67–5.05)	0.23
Female age	0.89 (0.73–1.08)	0.26
Male age	0.95 (0.86–1.05)	0.33
Female BMI (kg/m^2^)	0.90 (0.74–1.09)	0.31
AMH (pmol/L)	0.98 (0.93–1.03)	0.58
Stim Protocol	0.45 (0.17–1.20)	0.11
Trigger type	2.27 (0.16–31.79)	0.54
ICSI	1.09 (0.18–6.54)	0.92

Abbreviations: AMH, anti‐mullerian hormone; ICSI, Intracytoplasmic sperm injection.

### 3.2 Frozen Thawed Single Embryo Transfers

A total of 1294 single embryo transfers took place between January 2019 and December 2021. We observed a significant difference in the protocol for endometrial preparation, with proportionately more XX embryos transferred in a natural cycle (Table 4).

**TABLE 4:** Outcome data for transferred euploid embryos. Values are expressed as mean ± standard deviation.

	XX (*n* = 590)	XY (*n* = 642)	*p*
Endometrium preparation			
Natural thaw	275 (46.6%)	259 (40.3%)	0.019
Artificial thaw	162 (27.5%)	222 (34.6%)	
FSH thaw	153 (25.9%)	161 (25.1%)	
Endometrial thickness (mm)	9.2 ± 1.7	9.3 ± 1.6	0.24
Embryo developmental stage			
Day 5	199 (33.7%)	234 (36.4%)	0.55
Day 6	360 (61.0%)	379 (59.0%)	
Day 7	31 (5.3%)	29 (4.5%)	
High‐quality embryos^a^	569 (96.4%)	628 (97.8%)	0.14
Biochemical pregnancy per transfer	349 (59.2%)	395 (61.5%)	0.41
Clinical pregnancy per transfer	255 (43.2%)	266 (41.4%)	0.56
Miscarriage per clinical pregnancy	29/255	27/266	0.65
Live birth per transfer	223 (37.8%)	228 (35.5%)	0.40
Gestational age	38.1 ± 2.1	38.0 ± 1.6	0.76
LSCS per live birth	85/223 (38.1%)	107/228 (46.9%)	0.06
Birthweight (g)	3190 ± 779	3250 ± 809	0.47

Abbreviations: FSH, Follicle stimulating hormone; LSCS, Lower segment Caesarean section.

^a^ Defined as 3BB or better.

The sex ratio for thawed blastocysts was 1.09, with a ratio of 1.10 for blastocysts surviving the thaw process (Table 1).

There was no statistical difference in biochemical pregnancy rate (*p* = 0.41), clinical pregnancy rate (*p* = 0.56), miscarriage rate (*p* = 0.65) or live birth rate (*p* = 0.40) based on embryo sex (Table 4). The SSR was 1.02. There were no differences in endometrial thickness, embryo developmental stage or quality related to blastocyst sex (Table 4). Further, there were no differences observed in gestational age, mode of birth or birthweight related to blastocyst sex (Table 4).

## 4 Discussion

Our study has shown that the overall BSR was significantly skewed towards male sex, although this effect was lost when determining the euploid BSR. In the context of ART, we found no relationship between the BSR and baseline characteristics, stimulation protocols or fertilisation method. On multivariate logistic regression analysis, we found no association between euploidy and sex, or any other baseline or stimulation variables examined. We found no differences in the sex ratio between transferred blastocysts and the SSR.

Although not a true representation of the PSR, our BSR data lend support to the hypothesis of a male‐favoured sex ratio at conception [5, 23]. A male‐favoured BSR has recently been reported in a smaller study by Carrasco et al. [24], who noted a greater proportion of aneuploid male blastocysts. Even though the study by Orzack et al. [20] reported no difference in the cleavage‐stage/blastocyst composite sex ratio, they reported a higher rate of aneuploidy in male embryos.

Unlike in Carrasco et al. [24] in which fertilisation was purely by ICSI, our dataset includes oocytes fertilised via both IVF and ICSI. Given the male‐favoured BSR in their study, Carrasco et al. [24] hypothesised that the likely true PSR was 1 with pre‐biopsy loss of a greater proportion of female embryos. There is a lack of consensus in the literature however, with another recent study, also using solely ICSI for fertilisation, reporting a female‐favoured BSR with a higher proportion of aneuploid female embryos [25]. Other authors found no relationship between blastocyst sex and aneuploidy [26]. It is interesting to note that in our study, the driver for the male BSR appears to be blastocysts formed by conventional IVF. However, the large confidence interval for our adjusted odds ratio for fertilisation by ICSI highlights the uncertainty of our findings.

Our findings lend support to the hypothesis that PSR favours male sex at least in the context of ART. It is accepted that our findings concerning the BSR cannot necessarily be extrapolated to spontaneous conception. Indeed, it has been shown in both animal models [27] and humans [28] that embryos behave differently in vitro based on their sex, and this may or may not be replicated in utero.

Nevertheless, in the current study, the BSR and SSR were consistent with the SSR reported for the Australian population at birth in 2022 [29]. Our findings are also aligned with the Supramaniam et al. [10] study utilising HFEA data despite the fact that compared to Supramaniam et al. [10], our cohort differs in its higher proportion of fertilisation by ICSI (59.5% vs. 40.11%) and exclusive blastocyst transfer compared to 35.8% cleavage stage transfer. The relatively higher ICSI rate did not appear to lower the SSR as suggested by some groups [12], although it is possible that our inclusion criteria of blastocyst transfer only may be raising the ART SSR, in keeping with previous study findings [7, 9].

Looking at the transferred euploid embryos in our cohort, we found no sex‐related differences in biochemical pregnancy, pregnancy loss, live birth rate or relevant obstetric outcomes. The statistically significant difference in terms of endometrial preparation by sex is likely reflective of the retrospective nature of this project and has not had an impact on the downstream, clinically relevant outcome of biochemical pregnancy. Further, our secondary outcomes are corroborated by other recently published studies [24, 25].

The relative strengths of our study are not only the large number of embryos included but also the fact that it reflects modern ART practices, that is, a mixture of IVF and ICSI, blastocyst biopsy and blastocyst transfer. As with all retrospective studies, some of the drawbacks of our work are the potential issues with data entry and accuracy. Further, the data arise from patients undertaking PGT‐A for a specific reason, and this may introduce a degree of bias to our data. Finally, we did not examine whether the rate of embryo development or the day of embryo biopsy had an impact on embryo sex, as has been suggested by some studies [30].

There continues to be a lack of consensus on the impact of ART on sex ratios. Our study contributes to the body of evidence demonstrating no relationship between the BSR and euploidy, baseline characteristics, stimulation protocols or fertilisation method. We also found no differences in pregnancy outcomes based on embryo sex. Overall, our cohort's SSR was consistent with the SSR for the contemporary Australian population at birth, and various procedures and techniques used in ART do not appear to have a significant impact.

## Conflicts of Interest

The authors declare no conflicts of interest.
